# Spatial Analysis on Future Housing Markets: Economic Development and Housing Implications

**DOI:** 10.1155/2014/838021

**Published:** 2014-04-16

**Authors:** Xin Liu, Lizhe Wang

**Affiliations:** ^1^Yantai Institute of Coastal Research, Chinese Academy of Sciences, Yantai 264003, China; ^2^The University of Birmingham, West Midlands B15 2TT, UK; ^3^Institute of Remote Sensing and Digital Earth, Chinese Academy of Sciences, Beijing 100094, China

## Abstract

A coupled projection method combining formal modelling and other statistical techniques was developed to delineate the relationship between economic and social drivers for net new housing allocations. Using the example of employment growth in Tyne and Wear, UK, until 2016, the empirical analysis yields housing projections at the macro- and microspatial levels (e.g., region to subregion to elected ward levels). The results have important implications for the strategic planning of locations for housing and employment, demonstrating both intuitively and quantitatively how local economic developments affect housing demand.

## 1. Introduction

**Figure 1:**
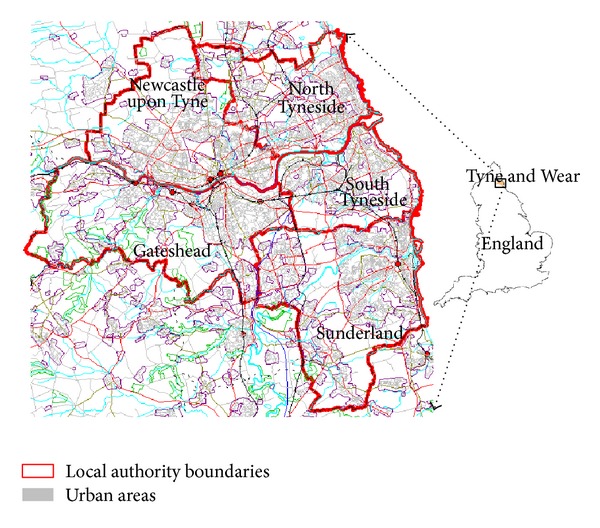
Location of the Tyne and Wear region.

The Tyne and Wear conurbation, located in the northeast region of the UK, has had a traditional coal mining economy since the 13th century (for geographic information, see Figure 1). Beginning in the mid-1970s, the UK economy has been significantly restructured [1]. A number of core themes are prominent in these changes, including a shift from primary and manufacturing industries to service sector employment [2]. As a result, Tyne and Wear experienced large-scale job losses in coal mining-related industries and the region became one of the most economically deprived areas in the UK. To boost local incomes and employment opportunities in the deprived area of Tyne and Wear, the central and local governments have been introducing a number of economic diversification and urban renaissance plans through enterprise and investment for decades.

As reviewed in the Annual Monitoring Report 2007, there are some notable improvements in Tyne and Wear. For example, the employment rate in Tyne and Wear rose over three times as fast as in England as a whole over the 1995–2006 period [3]. Recent improvements in local employment have prompted considerable interest in housing market response to economy development. To deliver a better fit between economic and housing aspirations in Tyne and Wear, a coupled projection methodology was employed in this paper. The newly developed method, based on a work carried out by Burfitt et al. [4], is capable of converting economic signals (e.g., employment) and forecasts into residential housing demands. The purpose of the study was to analyze the impact of economic change on residential housing and, given the nature of this interactive nexus, to estimate the rising demands on housing type and tenure. The results of this analysis could help local authorities facing budget constraints and strict sustainable housing targets to understand the possible changes in housing provision that may be required over a certain timeframe to achieve sustainable communities in the study area.

Housing constitutes a large share of the overall economy, and the significance of the housing market to the economy has been illustrated in many research papers [5, 6]. For example, detailed figures of the UK's wealth show that the most valuable asset in 2007 continues to be housing, with a total value of *£*4,314 billion, the equivalent of 62% of the nation's wealth in 2007 [7]. Moreover, housing is a key issue to consider in delivering healthy and attractive communities [8]. However, knowledge that is detailed enough to connect the housing market and economic analysis is scarce. A literature review of macroeconomics and housing conducted by Leung [9] indicated that the interactive nexus between the housing market and urban economics has been virtually ignored by conventional economics research.

The contribution of this study to the literature is fourfold. First, there is a small yet growing research effort that strives to understand the interplay between the housing market and economics [10–12]. However, to date, there has been no example of employing a quantitative method to delineate the impact of changes in the labor market (e.g., employment) on housing choice and residential mobility. The coupled projection method presented in this paper contributes to filling this gap by showing a quantitative estimation of likely housing demand driven by employment forecasts in Tyne and Wear. Second, no study has examined housing market response to economic development in Tyne and Wear. With the persistence of a rapid growth in employment rate, there is an urgent need for information on future housing demand in Tyne and Wear in order to develop sustainable communities and neighborhoods. Third, there is a relatively recent, growing recognition about the importance of the interactions between housing markets and the economy [9, 13–15]. This study, therefore, represents a timely opportunity to estimate the housing demand in Tyne and Wear. Finally, a similar model presented by Burfitt et al. [4] relies highly on economic forecast data for the subregions at the local authority level, both by standard occupation classification (SOC) and by standard industrial classification (SIC). Compared with the previous model, the coupled projection method requires fewer economic data requirements, indicating its highly potential applications in regional strategic planning.

## 2. The Investigation Region and Economic Data

As shown in Figure 1, the Tyne and Wear area in the northeast region of England covers five local authority districts (LADs): Newcastle upon Tyne, Gateshead, North Tyneside, South Tyneside, and Sunderland. These five LADs consist of a total of 113 electoral wards at the sublevel. Each local authority district delineates electoral wards that have a certain level of commonality in a number of neighborhoods, such as travel to work (TTW) pattern and accommodation utilization profile. These districts form a basis of this work and, along with the local wards, were taken as the key geography reporting scale throughout the study.

**Figure 2:**
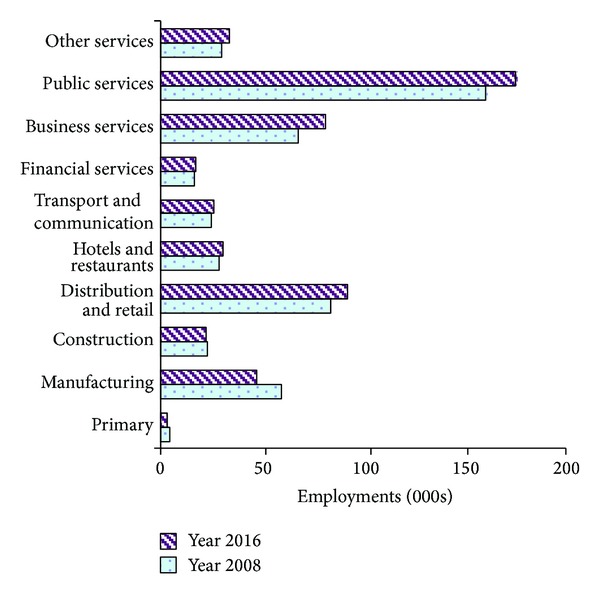
Anticipated employment by SIC (UK Standard Industrial Classification of Economic Activities) in the region of Tyne and Wear, 2008–2016. Source: TWRI.

The economic data underlying this report was provided by Tyne and Wear Research Information (TWRI). As shown in Figure 2, TWRI produces a forecast based on historical trends in the economic performance of Tyne and Wear. This forecast is the policy-neutral, or baseline, output of the regional econometric model. The forecast reflects long-term structure trends and assumes that they will continue into the next decade. All economic sectors, but primarily manufacturing and construction, will experience an increase in total employment over the 2008–2016 period. Among all sectors, net new employment in public services from 2008 to 2016 will be 5475 (34.9% in 2008), which will lead the public service sector to account for around one-third of total employment in the region. This trend is consistent with the national restructuring from a primary and industry economy to a services economy.

## 3. The Coupled Projection Methodology

**Figure 3:**
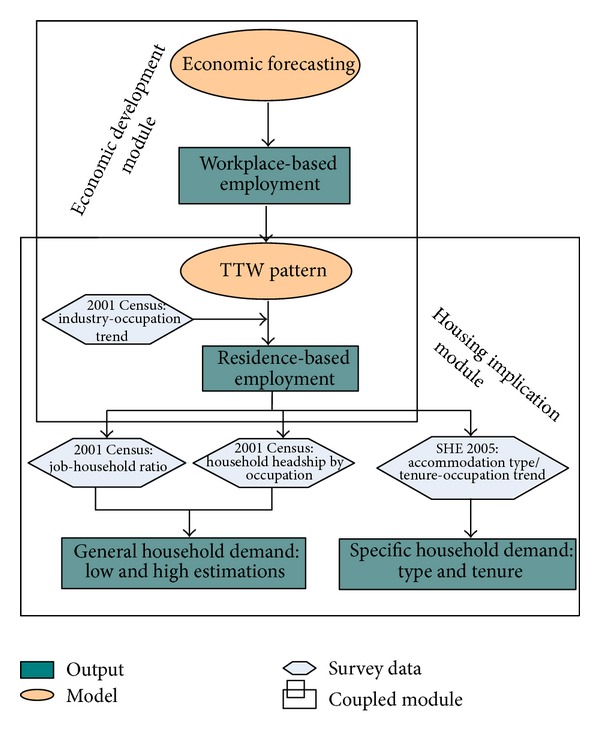
The coupled projection methodology. TTW: travel to work; SEH: Survey of English Housing.

The methodology used in projecting potential future housing demand is complex and draws on stages and sources, outlined in Figure 3. Two mappings are used to turn workplace-based employment forecasts by industry sector into residence-based employment. These mappings give the occupational structure of each industry and the TTW pattern of each occupation, respectively. The 2001 Census and the Survey of English Housing (SEH) 2005 provide information regarding job to employed household ratio, household headship rate by occupation, and housing types and tenures across the national housing market. Applying this information to the new residence-based employment forecasts allows an assessment of likely housing demand to be made. The detailed procedure of applying the coupled projection methodology into the Tyne and Wear case is described in the following paragraphs.

### 3.1. Industry-Occupational Mapping

The UK SOC consists of the following nine major groups: managers and senior officials; professional occupations; associate professional and technical occupations; administrative and secretarial occupations; skilled trades occupations; personal service occupations; sales and customer service occupations; process, plant, and machine operatives; and elementary occupations. The labor force survey provides information on recent trends in the occupational structure of each economic section (or SIC-SOC matrix). At this stage of the methodology, we assume that such trends will continue to 2016. According to the SIC-SOC matrix (IO_*ij*_) and relevant industry section forecasts, occupational outturns will be implied as follows:

(1) $$\begin{array}{c} \text{Oc}{\text{c}}_{j}=\overset{10}{\underset{i=1}{\sum }}\left(\text{Em}{\text{p}}_{i}*\text{I}{\text{O}}_{ij}\right);\;j=1,2,\ldots ,9, \end{array}$$

where Occ_*j*_ is the total number of employments for occupation *j*, Emp_*i*_ is the employment forecast in industry *I*, and IO_*ij*_ is the percentage of occupational employment *j* within section *i*.

### 3.2. Residence-Based Employment

In order to address the likely housing implications of employment growth in the region, it is necessary to convert economic forecasts (i.e., workplace-based employment) into residence-based employment. Based upon the prevailing TTW patterns generated from the 2001 Census [16], residence-based employment can be achieved as follows:

(2) $$\begin{array}{c} \text{R}{\text{E}}_{k}=\overset{9}{\underset{j=1}{\sum }}\left(\text{Oc}{\text{c}}_{j}*\text{TT}{\text{W}}_{jk}\right), \end{array}$$

where RE_*k*_ is the sum of residence-based workers in site *k* and TTW_*jk*_ is the ratio of residence to workplace-based workers with occupation *j* at site *k*.

### 3.3. Job-Related Household Projection

To estimate the likely impact of employment growth on household demand, a basic model with a ratio of jobs to households derived from the Labour Force Survey (LFS) [17] is used. Job-related household projection at site *k* (JHH_*k*_) usually proceeds through a process whereby the total job figure at site *k* (i.e., RE_*k*_) is divided by the general ratio of job to employed household (*a*):

(3) $$\begin{array}{c} \text{JH}{\text{H}}_{k}=\frac{\text{R}{\text{E}}_{k}}{a}. \end{array}$$

Clearly, this projection model is an oversimplification to estimate the likely pattern of household demand, as it treats all jobs the same, with the same capacity to form a household.

### 3.4. Job Type-Related Household Projection

There is clearly no one-to-one relationship between job creation and household demand, as described in Section 3.3. Many jobs are likely to be taken as second or third jobs within a household and, therefore, have reduced housing impact in terms of household formation [4]. Exploring in detail the relationships between different job types and housing allows for an accurate assessment of the potential of each job type to generate housing demand. In this way, job type-related household projection operates as a proxy measure for a job-related household model. More specifically, households formed in site *k* (OHH_*k*_) are aggregated as

(4) $$\begin{array}{c} \text{OH}{\text{H}}_{k}=\overset{9}{\underset{j=1}{\sum }}\left(\text{R}{\text{E}}_{jk}*\text{H}{\text{S}}_{j}\right), \end{array}$$

where RE_*jk*_ is workers with occupation *j* at site *k* and HS_*j*_ is the household headship rate by occupation *j*. Clearly, the application of the job type-related household model reduces the level of total household demand when compared to the aggregate figure based on the job to household ratio in Section 3.3 [4].

### 3.5. Housing Demand by Type and Tenure

The anticipated patterns of housing demand by type and tenure can be further examined by utilizing the aggregate household projection generated in Section 3.4 and taking current region housing profiles into account. Analysis of the SEH 2005 [18] dataset provides a brief profile of the current pattern of household consumption by occupation. In agreement with housing type and tenure variables defined in SHE [18], housing type consists of two main categories: house and flat. According to tenure, properties can be classified into owner occupied, social rented, and private rented. Property formation with type *m* and tenure *n* in site *k* can be estimated as

(5) $$\begin{array}{c} H_{mn}^{k}=\overset{9}{\underset{j=1}{\sum }}\left(\text{R}{\text{E}}_{jk}*\text{H}{\text{S}}_{j}*\text{T}{\text{T}}_{mn}^{j}\right), \end{array}$$

where *H*_*mn*_^*k*^ represents property with type *m* and tenure *n* at site *k* and TT_*mn*_^*j*^, a parameter derived from SHE [18], represents the percentage of specific property with type *m* and tenure *n* taken by occupational group *j*.

## 4. Results

### 4.1. Regional Housing Demand

**Figure 4:**
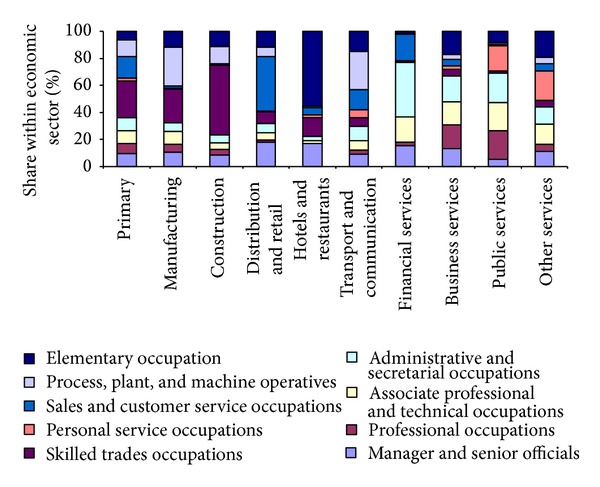
Occupational breakdown within economic sector in the Tyne and Wear region based on Office for National Statistics [7].

**Figure 5:**
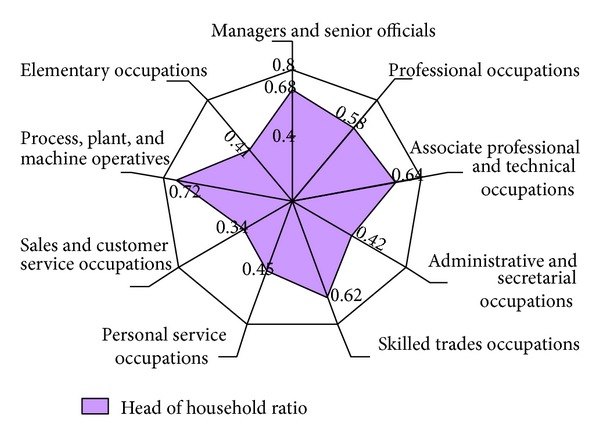
Estimated household headship by occupations, based on Labour Force Survey 2005 [17].

The following estimations, presented in Figure 4, Table 1, and Figure 5, are parameters estimated from Office for National Statistics [7], LFS household 2005, and LFS individual 2005 [17], respectively. These estimations are critical for converting workplace-based employment forecasts to households. Figure 4 maps trends in the occupational structure of each economic sector. Clearly, elementary occupations dominate the sector of hotels and restaurants, while construction is dominated by skilled service occupations. Table 1 sets out the key statistics on the relationship between employment and housing in the Tyne and Wear region. Working-age households represent 72% of the household consumption across the region, which is obviously higher than the employed households, as the former group includes households in which no one is employed, while, by definition, employed households have at least one person working. Employed households account for around 54% of all households. In other words, 46% of household consumption is not directly related to employment. There is an average of 1.85 people in employment in each employed household, meaning that there is a ratio of roughly 1.85 employees to every employed household. Therefore, at the simplified level, we might expect the net new employment growth identified in the TWRI scenario to generate new household demand at the rate of one household for every 1.85 net new jobs.

**Figure 6:**
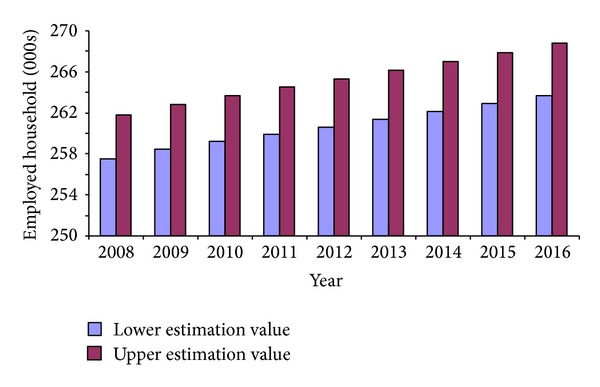
Projected household demand, 2008–2016. The lower estimation figure is based on head of household ratio by occupation, while the upper estimation figure is based on job to employed household ratio.

As mentioned in the previous section, another way to link employment with households is to identify the propensity for different job types to produce workers with household headship. Head of household broken down by worker occupation for the region is shown in Figure 5. Workers in occupations such as managers, skilled trade occupations, and machine operators have the highest headship rates. In contrast, workers in occupations associated with administrative, personal service, sales, and elementary (unskilled) activities are less likely to be heads of household. Following on from the job to employed household ratio and headship rate analysis, different fundamental implications for rates of household demand are shown in Figure 6. The lower estimation value is produced from the job type-related projection and the upper estimation is generated by applying the less specific job-related projection. Compared to the aggregate figure based on the job to employed household ratio, the application of headship rates by job types reduces the level of total household demand. This result can be attributed to the fact that new employment forecasts are based on occupations with lower propensities and capacities to form households than the current occupation profile of the region. Therefore, it is suggested that both estimations could be used to generate ranges of household demand for the region. The lower figure might be taken as an approximate estimation, while the upper figure is regarded as a conservative value for housing demand.

An outcome based on conservative estimation is that gross housing demand alongside employment growth accounts for 263,664 in 2016. If we look at net housing growth from 2008 to 2016, we conservatively have a total new demand of 6,119 households for the whole region.

### 4.2. Housing Demand at the Ward Level

Conservative estimations set out in the previous section can be further broken down spatially to examine housing demand at both the local authority district level and the ward level.

**Figure 7:**
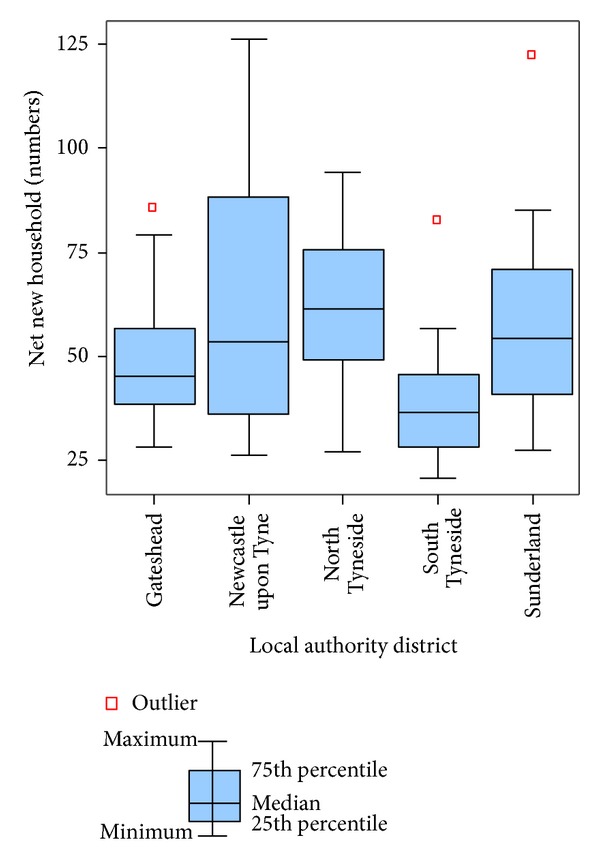
Aggregation of ward data on net new households: 2008–2016, by local authority districts.

**Figure 8:**
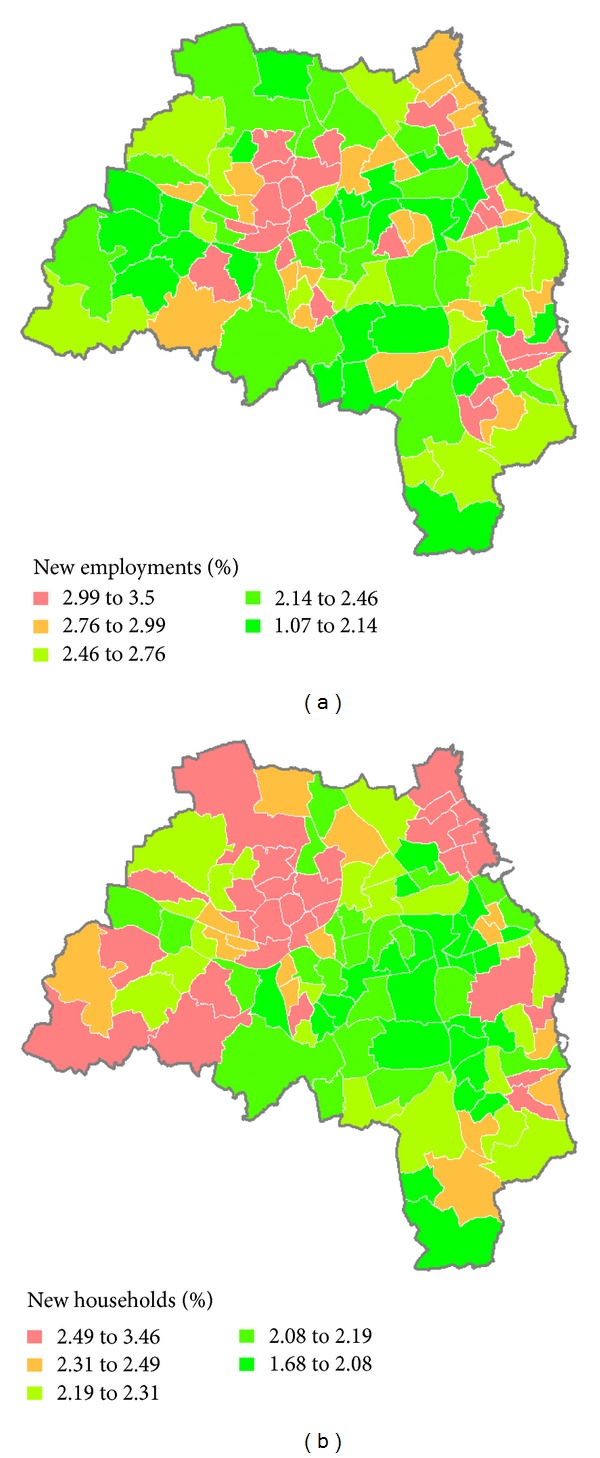
Household change by wards alongside employment growth, from 2008–2016. (a) Employment change (%) and (b) household change (%).

Figure 7 shows that new housing formation is more polarized in Newcastle upon Tyne, as both its range and the difference between the lower and upper quartile represent the highest value among the five LADs, while South Tyneside is likely to experience the lowest rates of increased housing demand in the region. The spatial distribution of potential housing demand driven by employment is shown in Figure 8(b). Central areas in the region highlighted in green in Figure 8(b) are forecasted to experience the lowest rates of demand for employment-related housing. Some wards in Newcastle upon Tyne, Gateshead, and North Tyneside marked with red are expected to experience strong growth of new housing. Compared with employment change, shown in Figure 8(a), this forecast suggests that patterns of household demand will not directly follow from employment creation. This finding can be attributed to the fact that there are distinct neighborhoods in these wards where the community will take up jobs with low rates of household formation. Equally, there are neighborhoods where workers are likely to take up jobs with higher rates of household formation. Under such circumstances, a more spatially differentiated pattern of household demand is produced. In order to provide sustainable housing, the government could be aware of the availability of housing premises in local wards to meet the needs of new households in 2016.

**Figure 9:**
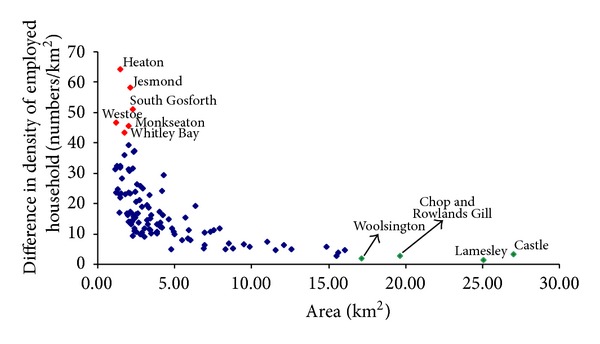
Forecast change in density of employed household by ward, 2008–2016. Particular changes over 40 households/km^2^ are highlighted by red spots, together with the ward name. The green marks, with their corresponding ward names, represent the four largest wards with little change in density of employed households from 2008 to 2016.

To determine the polarized pattern of housing growth in the region, the change in density of employed households in all wards within the region was examined.  Figure 9 shows the shift in employed household densities for all wards against their geographic areas during the 2008–2016 period. Ten particular wards, including the six most significantly changing wards and the four most underperforming wards with the largest geographic areas, are estimated in the region, as shown in Figure 9. The red marks are wards where new housing is expected to grow significantly more rapidly than in others. On the other hand, they could be regarded as the most preferred living locations for new employed households. The green markers represent wards with little change in density of employed households. To an extent, they do not comprise the competitive housing market, and thus they are estimated to be the least preferred housing locations for new households. Policy makers would need to consider improving the neighborhoods to attract more people to move to these areas in the future.

### 4.3. Accommodation Type and Tenure

**Figure 10:**
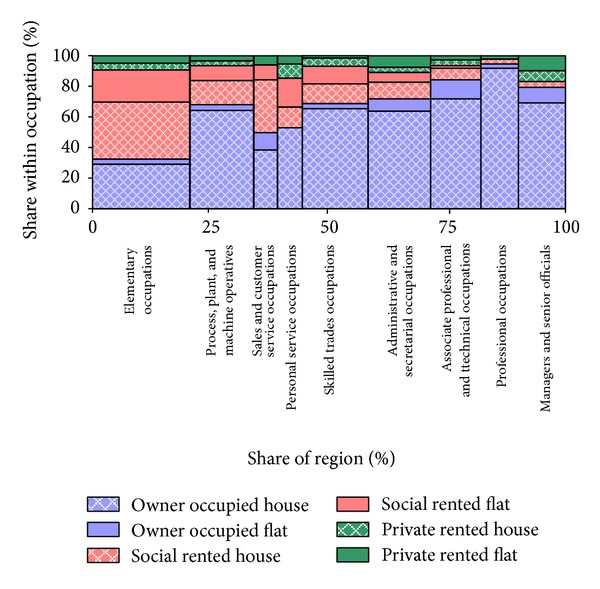
Type and tenure utilization breakdown by occupation in the Tyne and Wear region based on Labour Force Survey 2005 [17].

A more detailed analysis of the likely nature of occupation-related housing demand is provided in this section.  Figure 10 depicts an overall regional profile of accommodation type and tenure utilization by occupation in 2005. A marked difference between accommodation type and tenure utilization can be seen. Of these, the vast majority are some form of owner occupied housing, followed by social rented housing. Heads of household with elementary occupations make up 22% of employed households and are dominant in the region. Based on the current pattern of household consumption by occupation and future forecasts by occupation, Table 2 provides an analysis of the volume of housing demand arising from the future scenario of forecasts by occupation. Table 2 shows a markedly different spatial pattern of demand in both volume and type, with a considerable rise in extra demand for owner occupied properties in Newcastle upon Tyne. Among the five local authority districts, the extra demand for social rented properties is the highest in Sunderland. Private rented properties also increase substantially across all local authority districts in the region. However, owner occupied houses comprise nearly 60% (3385) of newly formed households.

## 5. Conclusions and Policy Implications

In this paper, a coupled projection method was developed to estimate likely housing demand, including locations, types, and tenures of new properties, if the economic employment forecasts are translated into reality. Given that new employments in Tyne and Wear through 2016 are forecast to number 12,894, six individual wards, including Heaton, Jesmond, South Gosforth, Westoe, Monkseaton, and Whitley Bay, are estimated to be the most preferred locations by new employed households. By contrast, Woolsington, Chop and Rowlands Gill, Lamesley, and Castle are forecasted to be the least preferred locations for new households to settle. This is only a “policy off” context or baseline scenario, that is, what we would expect to happen if we assume that the historical economic employment trend persists until 2016. In order to conduct effective regional planning, local authorities would need to compare the baseline option with a “policy on” scenario, in which economic employment with aspiration is delivered and used again by the couple projection model to produce the anticipated impacts on future housing demand.

Overall, the method developed in this paper is straightforward. It is capable of combining both an economic employment model and geographic information system knowledge and statistical technique. These benefits and the model's application potential in the field of regional strategic planning have been demonstrated using the case of Tyne and Wear presented in this paper.

## Figures and Tables

**Table 1:** Key employment and household data in Tyne and Wear.

	Tyne and Wear
Total households	473,261
Average number of people per household	2.21

Total working-age households	340,975
Working-age households as share of total households	72%
Average number of people in work in working-age households	1.27

Total employed households	257,602
Employed households as a share of total households	54.4%
Average number of employed people per employed households	1.71

Total jobs (main and second jobs)	476,922
Jobs to working-age households ratio	1.40
Jobs to employed households ratio	1.85

(Source: LFS, 2005 [17] and SEH [18]).

**Table 2:** Net additional housing demand by local authority district, 2008–2016.

Local authority district	Owner occupied		Social rented		Private rented	
	House	Flat	House	Flat	House	Flat
Gateshead	609	85	151	72	41	64
Newcastle upon Tyne	1074	119	197	93	56	87
North Tyneside	799	98	152	72	57	72
South Tyneside	472	61	105	51	29	46
Sunderland	861	115	212	100	55	87

Total (numbers)	3855	478	817	387	228	356
